# Comparative Analysis of Hydrogel Adsorption/Desorption with and without Surfactants

**DOI:** 10.3390/gels10040251

**Published:** 2024-04-08

**Authors:** Salam Abdulla Dhahir, Auda Jabbar Braihi, Salih Abbas Habeeb

**Affiliations:** Polymer and Petrochemical Engineering Department, College of Engineering Materials, University of Babylon, Babylon 51002, Iraq

**Keywords:** pullulan hydrogel, adsorption/desorption, heavy metal ions, reusability, adsorption kinetic models

## Abstract

In this particular study, a hydrogel known as SAP-1 was synthesized through the grafting of acrylic acid-co-acrylamide onto pullulan, resulting in the creation of Pul-g-Poly (acrylic acid-co-acrylamide). Additionally, a sponge hydrogel named SAP-2 was prepared by incorporating the surfactant sodium dodecyl benzene sulfonate (SDBS) into the hydrogel through free radical solution polymerization. To gain further insight into the composition and properties of the hydrogels, various techniques, such as Fourier transform infrared spectroscopy, hydrogen nuclear magnetic resonance (1H NMR), atomic absorption spectroscopy, and field emission scanning electron microscopy (FE-SEM), were employed. Conversely, the absorption kinetics and the equilibrium capacities of the prepared hydrogels were investigated and analyzed. The outcomes of the investigation indicated that each of the synthesized hydrogels exhibited considerable efficacy as adsorbents for cadmium (II), copper (II), and nickel (II) ions. In particular, SAP-2 gel displayed a remarkable cadmium (II) ion absorption ability, with a rate of 190.72 mg/g. Following closely, SAP-1 gel demonstrated the ability to absorb cadmium (II) ions at a rate of 146.9 mg/g and copper (II) ions at a rate of 154 mg/g. Notably, SAP-2 hydrogel demonstrated the ability to repeat the adsorption–desorption cycles three times for cadmium (II) ions, resulting in absorption capacities of 190.72 mg/g, 100.43 mg/g, and 19.64 mg/g for the first, second, and third cycles, respectively. Thus, based on the abovementioned results, it can be concluded that all the synthesized hydrogels possess promising potential as suitable candidates for the adsorption and desorption of cadmium (II), copper (II), and nickel (II) ions.

## 1. Introduction

Hydrogels are known as hydrophilic, insoluble polymers with a high swelling capacity due to their three-dimensional networks, enhancing their ability to absorb large amounts of aqueous solutions. The hydrophilic property of the hydrogel could be taken advantage of by grafting the basic unit of the hydrogel with active groups such as carboxyl groups (–COOH) and amide (–CONH_2_) to increase the absorption capacity of the hydrogel [1]. Hydrogels are often used in many industrial, agricultural [2], biomedical [3], and wastewater treatment applications [4,5]. Pullulan is a non-toxic polysaccharide, as well as being hygroscopic, edible, biocompatible, biodegradable, and water-soluble, produced by the yeast Aureobasidium [6]. Pullulan is characterized by its ability to absorb water, in addition to its excellent adhesive properties that ensure its efficiency in the formulation of membranes and fibers. It is used in various fields, such as agriculture, the food industry [7,8], pharmaceuticals [9], and textiles [10]. Pullulan consists of repeated α-(1,6) maltotriose units) via α-(1,4) glycosidic bonds and has the chemical formula (C_6_H_10_O_5_)n [11]. Pullulan, in its natural state, is not used in wastewater treatment because it is soluble in water, but the procedure of the graft polymerization of acrylamide and acrylic acid, used as monomers, enhances the strength of the hydrogel and makes the hydrogel insoluble in water [12,13]. The industrial and domestic applications of surfactants have increased due to their versatility and structural diversity [14]. Surfactants are divided according to the charge of the main group into four types: anionic, non-ionic, cationic, and amphoteric [15]. The most important processes of surfactants’ interactions with polymers are precipitation, complexation, and gelation in solution. The origins of the interaction between polymers and surfactants, such as electrostatic dipoles, hydrophobicity, and hydrogen bonding, differ [16]. These materials work to change hydrophobicity to hydrophilicity [17,18]. In addition, these materials work to improve the mechanical ability of polymers and their interfacial properties and improve the adsorption behavior of polymers [19,20,21,22,23] by intercalating the surface of non-ionic polymers with an ionic reducing agent. Such surfactants include sodium dodecyl benzenesulfonate (SDBS) [24]. Recent studies have focused on heavy metal ions as important environmental pollutants that should receive widespread attention [25]. The increase in industrial processes, such as the production of batteries, artificial leather, electroplating, and textiles, has contributed to the increase of these pollutants in surface water, drinking water, and even groundwater [26]. Research studies indicate that cadmium ion pollutants cause breast and prostate cancer [27], in addition to affecting the body’s health, especially that of the liver tissue [28], and increasing the development of bone and muscle diseases [29]. As for copper ion pollution, it has toxic effects on the environment and fish farming [30].

The health effects caused by swallowing or consuming nickel are pulmonary fibrosis, kidney disease, and cancer, in addition to cardiovascular disease [31]. This is considered a major pollutant of water bodies [32] and affects the liver and brain [33]. Therefore, it has become mandatory to dispose of industrial wastes containing cadmium (II), copper (II), and nickel (II) ions, and their removal from aqueous solutions has received wide attention [34]. Ion exchange and chemical and electrochemical precipitation are traditional processes for removing these pollutants from wastewater. However, they have some disadvantages, such as the need for high amounts of energy and corrosion [35]. Fome et al. used the MOF-88 particles to reinforce the polyacrylonitrile nanofiber membranes to remove Pb^2+^ ions from aqueous solutions. Filtration membranes were produced that can produce more than 500 L of pure water per square meter of the membrane after purifying the water from heavy metal ions such as Zn^2+^, Cd^2+^, Pb^2+^, and Hg^2+^ [36].

A cation exchange membrane was manufactured, consisting of PVC-co-2, acryla-med-2-methylpropane, and sulfonic acid with a separation capacity of 99.9%, 99.9%, and 96.9% for K^+^, Pb^2+^, and Ni^2+^ ions, respectively, by Nemati et al. [37]. On the other hand, using the biomass of Avena fatua, a non-toxic weed that grows easily and in large quantities for the biosorption of cadmium (II), copper (II), lead (II), and zinc (II) ions from aqueous solutions was developed by Arico et al. In addition, the effect of cadmium (II), copper (II), lead (II), and zinc (II) ions on the growth of A. fatura was studied [38]. Hydrogels are considered one of the most useful adsorbents due to their hydrophilic structure and ability to remove various types of contaminants. Therefore, Rady et al. were able to prepare a non-toxic porphyrin-silica chelate particle to adsorb cadmium (II), copper (II), lead (II), and zinc (II) ions from aqueous solutions. Adsorption was found to reach equilibrium after 25 min [39]. This study aims to prepare pullulan-g-poly (AA-co-AAm) (SAP-1) and (acrylic acid-co-acrylamide) containing an anionic surfactant as the sodium dodecyl benzene sulfonate (SAP-2), synthesized by free radical solution polymerization for the removal of cadmium (II), copper (II), and nickel (II) ions from aqueous solutions. The hydrogels’ experimental equilibrium capacities, their adsorption kinetics models, their metal ion adsorption and desorption rates, and their reusability were investigated throughout this study.

## 2. Results and Discussion

### 2.1. Chemical Structure Justification

**Figure 1:**
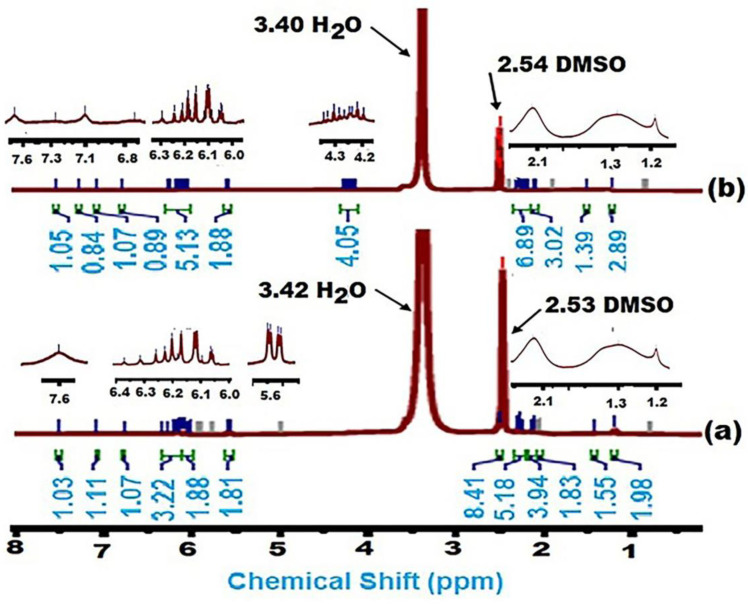
^1^H NMR spectra of (**a**) SAP-1, and (**b**) SAP-2.

Figure 1 shows that analysis by ^1^H NMR spectroscopy further supported the success of the SDBS modification of (pullulan-g-poly (AA-co-AAm)). In addition to the different carbons appearing at around 5.575–5.675 and 6.01–6.42, representing the Cis portion and trans portion of polyacrylic acid, those of the SAP-1 were converted to 5.590–5.685 and 5.99–6.28 ppm; these results agree with a previous study [40]. In addition, the new chemical shifts for protons in the range of 0.85–1.5 ppm represented the pullulan, which converts to 0.85–1.52 after adding the SDBS at SAP-2, in addition to the peak at 2.53 ppm, characterized by the methylene groups(–CH_2_CH_2_–) and the range of peaks (6–7 ppm) for amino groups that convert to 2.54 ppm and 6–7.6 ppm after adding the SDBS. These results agreed with previous studies [41,42].

**Figure 2:**
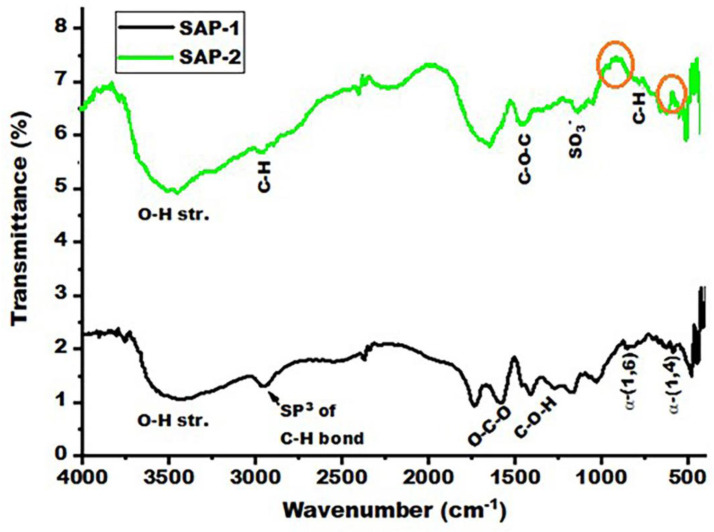
FT-IR spectrum of SAP-1 and SAP-2.

Figure 2 (SAP-1) shows that acrylic acid and acrylamide are grafted into pullulan. The appearance of bands from about 1707 and 1645 cm^−1^ indicates AA grafting, where AA contains a carboxyl group (COOH) [43]. This group includes the OH and C=O groups, which usually appear in these positions. The AM grafting that occurs in the bands at about 1000–1250 cm^−1^ and the bands at about 3217.71–3425.58 cm^−1^ represents the symmetrical and asymmetrical stretching of the N–H group from AM and the O–H stretching from AA and Pullulan.

**Figure 3:**
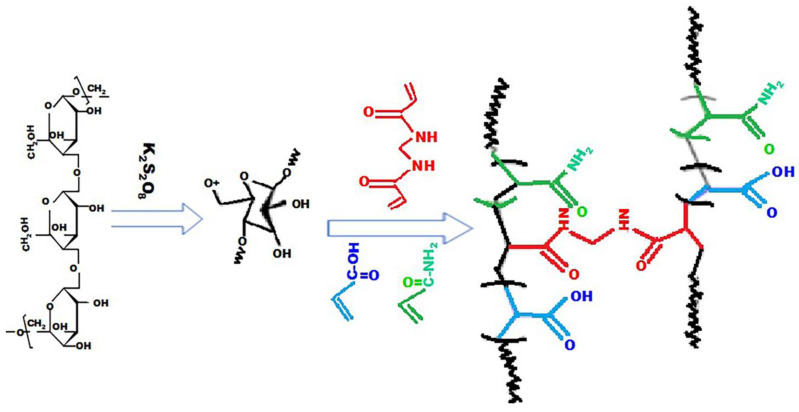
The schematic for the grafting of the acrylic acid and acrylamide into pullulan and the formation of the hydrogel (SAP-1).

The AM was grafted onto the PUL substrate through an aliphatic C–N bond, where its band appeared at 1000–1250 cm^−1^. In contrast, AA grafting occurred through a single C–C bond; however, since this bond is nonpolar (no difference in electronegativity), it usually does not show up as peaks in the IR spectrum. A covalent bond consists of two electrons from each of two carbon atoms. This is called a sigma bond (σ) between one orbital of each carbon atom. The peak is about 1658.71 cm^−1^ representing the stretching of the O–C–O group from Pullulan [12]. Peaks in the spectrum at 2852, 2927, and 2947 cm^−1^ are induced by C–H vibrations in SDBS at SAP-2. The absorption peaks at 3476 and 1187 cm^−1^ are attributed to the remaining –OH groups and C–O–C bonds on the SAP-2, and the peaks at 1163 are assigned to the ionic sulphonate SO^3−^ group present in SDBS [44,45]. The peaks of 887 and 1024 cm^−1^ did not appear in SAP-1 and appeared after adding the SDBS to the hydrogel, while the peaks at 1178 cm^−1^ and 2956 cm^−1^ represent the –COO– to extend the acrylate groups and the C=O extension of the acrylamide groups and C–H vibrations in SDBS, respectively, which indicates that a grafting copolymer reaction occurs on the pullulan backbone [46]. Therefore, grafting monomers such as acrylic acid and acrylamides onto pullulan leads to the formation of a three-dimensional network that contributes to increases in the adsorption of heavy metal ions by the surface of the hydrogels, as shown in Figure 3.

### 2.2. Adsorption Properties

#### 2.2.1. Adsorption Capacity

**Figure 4:**
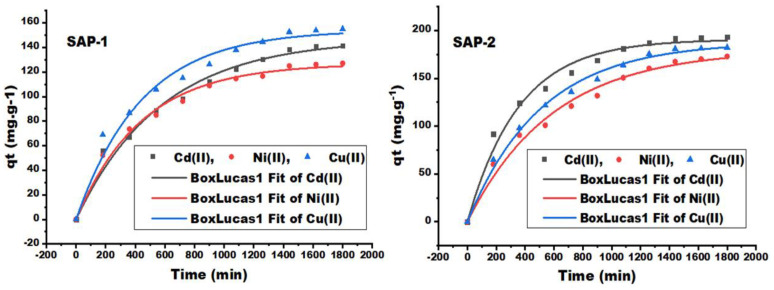
The adsorption capacity of heavy metal ions as Cd (II), Ni (II), and Cu (II) by hydrogels SAP-1 and SAP-2 at many different contact times.

Figure 4 shows the relationship between the absorption capacity of heavy metal ions, such as cadmium, nickel, and copper ions, from their aqueous solutions by the prepared hydrogels and a contact time of up to 1800 min. Using Equation (1), the absorption capacity is calculated at any contact time (mg/g), as the absorption capacity gradually increases until it finally reaches a plateau trend, with a non-linear relationship with contact time and for all heavy metal ions using prepared hydrogels. This demonstrates that the active sites of the adsorbent were gradually saturated and approached their maximum adsorption capacity. The maximum adsorbent capacity by SAP-1 was 154 and 146.9 (mg/g) for Cu (II) and Cd (II), while the maximum adsorbent capacity by SAP-2 was 190.72 and 187.978 (mg/g). The above results indicated a good interaction between the Cu (II) and Cd (II) and the hydrogel surface. The prepared gels can absorb all metal ions under investigation due to their carboxyl, amine, and hydroxyl functions, which were grafted onto the pullulan spine through acrylic acid and acrylamide [47,48,49,50].

However, the porous hydrogel has a greater effect, as the absorption capacity increases with the presence of SDBS, which causes an increase in the capacity and number of gaps within the structure of the hydrogels [12]. The removal efficiencies (%) of heavy metal ions from the acquiesce solution increased from 40.83 to 49.33% for Cd (II), from 43.21 to 54.0% for Cu (II), and from 42.77 to 47.69% for Ni (II) after adding the SDBS to hydrogel.

#### 2.2.2. Adsorption Mechanism Models 

**Figure 5:**
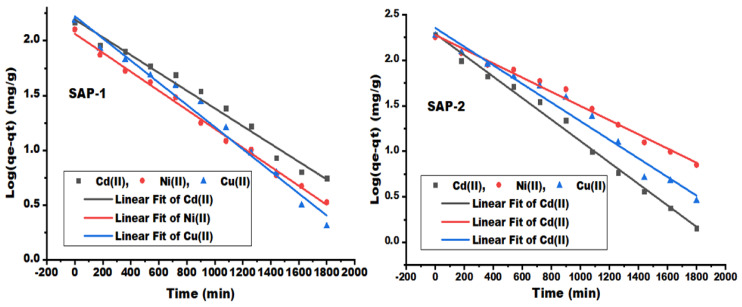
Pseudo-first-order kinetic model of heavy metal ions, such as Cd (II), Ni (II), and Cu (II), by hydrogels SAP-1 and SAP-2 at many different contact times.

**Figure 6:**
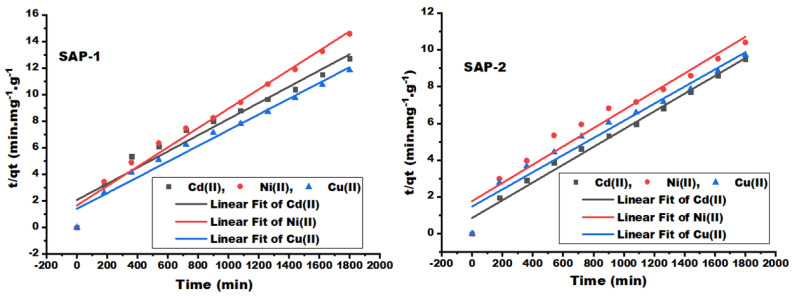
Pseudo-second order kinetic model of heavy metal ions, such as Cd (II), Ni (II), and Cu (II), by hydrogels as SAP-1 and SAP-2 at many contact times.

**Figure 7:**
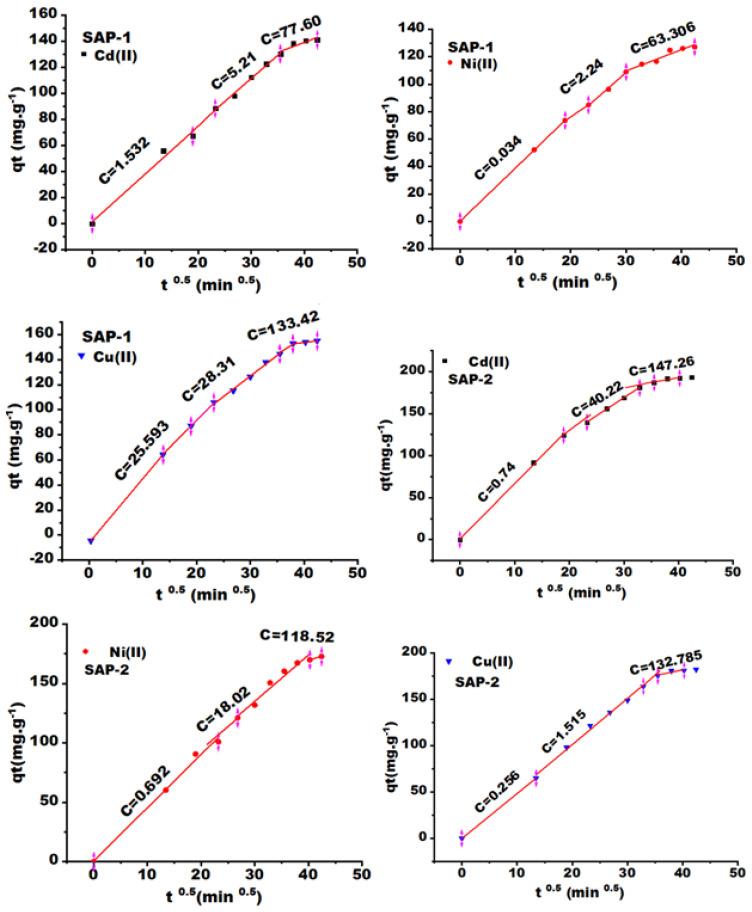
Weber–Morris kinetic model of heavy metal ions, such as Cd (II), Ni (II), and Cu (II), by hydrogels SAP-1 and SAP-2 at many different contact times.

The adsorption mechanism uses three steps: 1. transference of heavy metal ions to the adsorbent surface; 2. diffusion of heavy metal ion molecules into the adsorbent interior; 3. interaction of heavy metal ion molecules with reactive sites and the polymer structure (hydrogels) [15]. Figure 5, Figure 6 and Figure 7 represent three models for studying the adsorption kinetics of heavy metal ions from their aqueous solutions using prepared hydrogels, such as the pseudo-first-order, pseudo-second-order, and Weber–Morris kinetic model, for many different contact times. Figure 5 shows the relationship between the log (qe-qt) and contact time (t) in the first-order kinetic model, while the relationship between the t/qt and contact time (t) in the second-order kinetic model is shown in Figure 6. The second-order kinetic model was more regular and exhibited a linear relationship correction factor (R^2^ > 0.97) compared to the first-order kinetic model (R^2^ > 0.88) for both adsorbents. 

The theoretical equilibrium capacity of Cd (II) and Cu (II) at the second-order kinetic model were 206.61 and 214.59 mg/g for SAP-2, which indicated a good agreement with the experimental equilibrium capacities of 190.722 and 187.978 mg/g (the average results shown in Table 1, repeated three times). The adsorption rate constants were highly reduced in the second-order kinetic model (K_2_) compared to the adsorption rate constants in the first-order kinetic model (K_1_). This leads to a reduction in the reaction rate, which could be due to the decrease in adsorption sites on the adsorbent [12]. 

Due to the porous nature of the prepared gels, especially SAP-2, first- and second-order kinetic models are not suitable for explaining the adsorption mechanism. Therefore, the intraparticle diffusion model was also used to evaluate the relative importance of intraparticle diffusion. The theoretical equilibrium capacity (C) was determined according to Equation (5), which represents the interception plotted between the qt and t ^0.5^, while the intra-particle diffusion rate constant K_i_ represents the slope illustrated in Figure 7. The absorption kinetics of this model are divided into different steps, such as transferring the solute to the surface of absorbent particles, as well as transporting it from the surface of absorbent particles to active sites within the particle and then retaining it on these sites across the absorption and sedimentation phenomena within particles [15,51]. 

The curves of Figure 7 are divided into three steps: the first and second steps are the fastest in terms of the adsorption process, while heavy metal ion molecules work to penetrate the internal pores of the gel material in the third step, which leads to a gradual reduction in the adsorption rate, as shown in Table 2, where the diffusion rates within the particles are arranged as follows: ki1 > ki2 > ki3. In addition, the theoretical adsorption capacity (C) in the first stage is low, while the adsorption rate constant is high. However, after this, the slope of the curves was low, while C became high, indicating the pores are filled with particles of heavy metal ions in the final stages. The final stage is the equilibrium stage, which is slow, and diffusion into the pores or within the particles decreases. This can be attributed to the reduction in pore size, as well as the decrease in the concentration of adsorbents in the solution [52]. The theoretical adsorption capacities (C) of Cd (II) and Cu (II) at the final stages by SAP-2 and SAP-1 were 147.26, 132.785, 77.60, and 133.42 mg/g respectively.

### 2.3. Adsorption and Desorption Properties

#### 2.3.1. FE-SEM Analysis of Hydrogels 

The acrylic acid and acrylamide are grafted into pullulan in the form of (pullulan-g-poly (AA-co-AAm)), as shown in Figure 3. 

However, the addition of SDBS during the preparation of the hydrogel (SAP-1) leads to the formation of spherical micelles on the surface of the hydrogel. A super-absorbent spongy structure is created after removing the SDBS micelles through the washing process, as these micelles contribute to an increase in the absorption capacity of the hydrogel for heavy metal ions [53]. 

**Figure 8:**
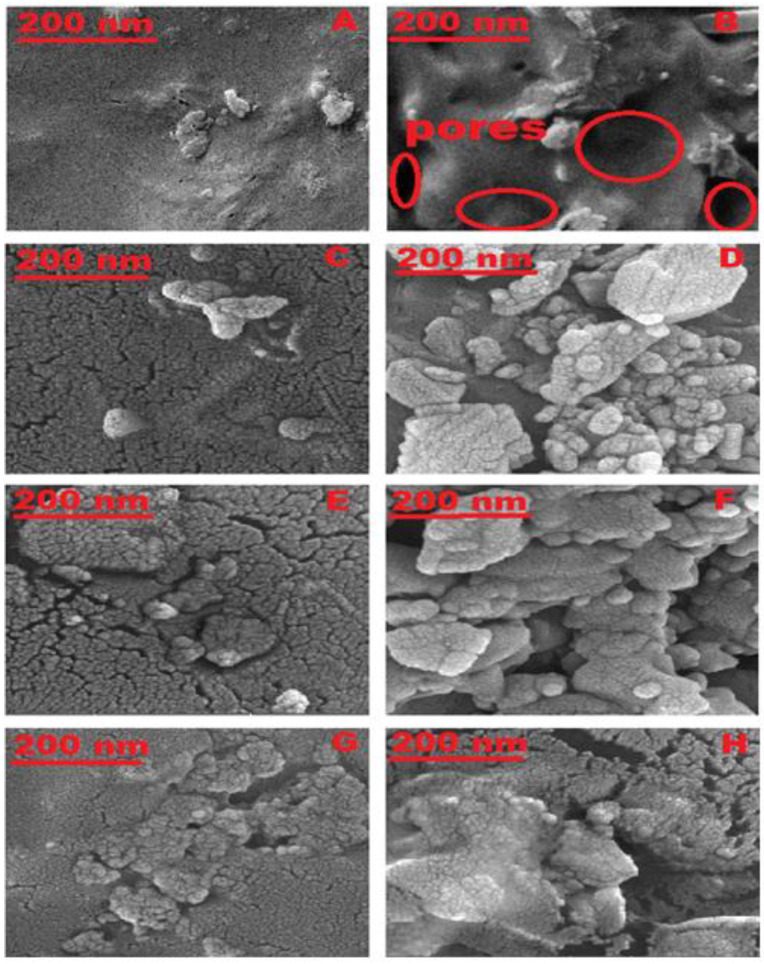
FE-SEM images of hydrogels before adsorption (**A**) (SAP-) and (**B**) (SAP-2), in addition to FE-SEM images of hydrogels after adsorption of (**C**) Cu (II), (**E**) Ni (II), and (**G**) Cd (II) by SAP-1 and (**D**) Cu (II), (**F**) Ni (II), and (**H**) Cd (II), by SAP-2, respectively.

Figure 8A,B shows the FE-SEM images of hydrogels before adsorption (A) (SAP-1) and (B) (SAP-2). On the other hand, it cannot reveal the general surface of the particles in samples before and after adsorption, but it can monitor the surface changes in the hydrogel before and after adsorption. The locations where the molecules were as similar as possible were identified. In terms of the shapes and dimensions before and after adsorption, the FE-SEM images showed that the surface of the hydrogel (SAP-1) was soft and had a lamellar structure before and after adsorption. As the surface of the hydrogel (SAP-2) became rougher, flocs appeared, and the pore size increased before and after adsorption, as shown in Figure 8C–H for hydrogels after the adsorption of Cu (II), Ni (II), and Cd (II), respectively.

#### 2.3.2. EDS Analysis of Hydrogel after Adsorbing/Desorbing

**Figure 9:**
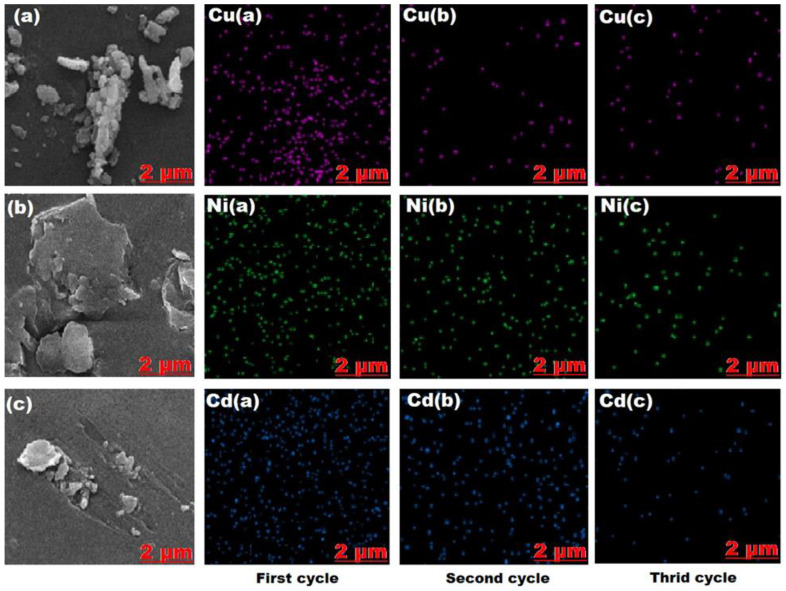
EDS scanning diagram of the original hydrogel SAP-2 after the desorption of (**a**) Cu (II), (**b**) Ni (II), and (**c**) Cd (II) in addition to the EDS scanning diagram of the hydrogel SAP-2 after desorption of the Cu (II), Ni (II), and Cd (II) in the first, second, and third cycles.

EDS examined the surface of the hydrogel samples (SAP-2) during adsorbing/desorbing. As can be seen from Figure 9, the original surface of the sample was mainly composed of C, N, O, Si, Cl, and Al elements. The dotted distribution of C was similar to that of rough surface areas in the tested (SAP-2) sample; this indicates the surface roughness of the hydrogel. The distribution of O, Al, and Si elements was uniform, but the order of the copper, nickel, and cadmium elements descended with an increase in the adsorbing/desorbing cycles, as shown by the EDS scanning mapping images in Figure 9, and the chemical elements remaining in the hydrogel (SAP-2) after the desorption of heavy metal ions for each cycle in Table 2. This result agreed with the previous study [53].

#### 2.3.3. Adsorption/Desorption Capacity 

**Figure 10:**
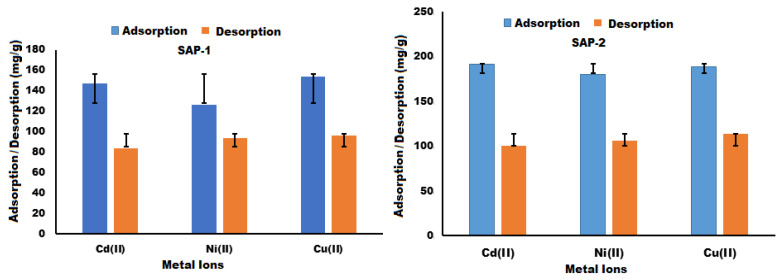
Adsorption/desorption capacity of heavy metal ions by hydrogels SAP-1 and SAP-2.

The adsorption and desorption capacities of hydrogels such as SAP-1 and SAP-2 were measured using an atomic absorption spectrometer (AAS-7000) for cadmium (II), copper (II), and nickel (II). The reason the hydrogels could adsorb all the elements under study is their carboxyl, amine, and hydroxyl functionalities, which are grafted onto the pullulan backbone through the addition of acrylic acid and acrylamide, which make these hydrogels good adsorption candidates for heavy metal ions [54,55,56,57,58,59,60]. The best-adsorbed metal ions were found to be Cd (II) and Cu (II) ions after 24 h; they were 190.72, 187.978, 146.9, and 154 (mg/g) respectively, which can be seen in Figure 4 and Figure 10. 

Desorption studies were performed by immersing the hydrogel-carrying heavy metal ions, such as cadmium (II), copper (II), and nickel (II), in 0.1 M HCl for 24 h. After 24 h, the desorption rates of cadmium (II) and copper (II) ions were found to be 100.43, 113.68, 84.25, and 96.05 mg/g, respectively, as seen in Figure 10. This study used hydrochloric acid to adsorb/desorb metal ions without applying any external forces. The desorption of metal ions can be enhanced using external forces such as heating. In addition, homogeneously distributed active sites on the hydrogel pore surfaces lead to an increase in the absorption capacity of the porous gels compared to the absence of a surfactant such as SDBS in the gel [61], as (R^2^ of SAP-2 > R^2^ of SAP-1) and the error bar (%) is lower than that in SAP-1. Therefore, in the HCL solution, the protons compete with metal ions for the carboxyl groups, which are responsible for the easy desorption of metal ions. The ability of the hydrogels prepared in this study to adsorb metal ions was compared with that of other adsorbent hydrogels in the literature, as shown in Table 3.

#### 2.3.4. Reusability

**Figure 11:**
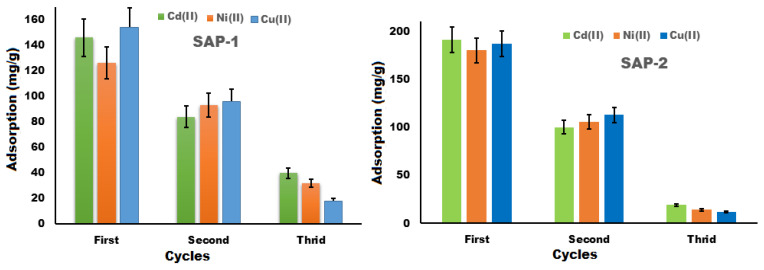
Relationship between the ability of hydrogels to reusable heavy metal ions such as copper (II), cadmium (II), and nickel (II) ions over three cycles.

One of the most important advantages of the adsorbent is its reusability, which makes it more cost-effective as it is able to remove most metal ions from wastewater. Hydrogels were used for three adsorption-desorption cycles to remove heavy metal ions such as cadmium (II), nickel (II), and copper (II) [43]. Figure 11 shows the adsorption amounts of heavy metal ions for three successive cycles, where the SAP-2 hydrogel was able to absorb a variety of ions with a greater adsorption capacity than the SAP-1 hydrogel. Because the SAP-2’s ability to swell is greater than that of SAP-1, the interaction of metal ions with functional groups is easier compared to the interaction of metal ions with functional groups in SAP-1. In addition, both hydrogels contain two monomers and have more functional groups available to absorb and reuse different metal ions. In general, the prepared gels can absorb copper (II) and cadmium (II) ions more than once, and better than nickel ions.

## 3. Conclusions

Free radical polymerization was used to produce the prepared hydrogels in this study. Many essential properties were used to determine the efficiency of the prepared hydrogels in absorbing heavy metal ions from aqueous solutions, including Fourier transform infrared spectroscopy (FTIR), hydrogen nuclear magnetic resonance (1H NMR), atomic absorption spectroscopy, and field emission scanning electron microscopy (FE-SEM), to further understand the composition and properties of the hydrogels, in addition to conducting an analysis of all models of absorption kinetics and choosing an appropriate model for the behavior of the prepared gels. After conducting a study on the reuse of the adsorbent material three times, it was found that the two gels are both good candidates for the adsorption of heavy metal ions, but the SAP-2 hydrogel is better, especially for the adsorption of both cadmium and copper ions. In general, the adsorption capacity of the prepared hydrogels tends to decrease with an increase in the number of adsorption cycles, and the prepared hydrogels have the ability to adsorb more than one metal ion.

## 4. Materials and Methods

### 4.1. Materials

To prepare the SAP-1 hydrogel, pullulan (PUL) and potassium persulfate, used as an initiator, were supplied by (Sigma-Aldrich, Burlington, MA, USA). In contrast, N,N-methylene bisacrylamide (MBA) was used to form crosslinks, and the monomers used to prepare the gel, such as acrylic acid and acrylamide, were supplied by (Merck, Darmstadt, Germany). Sodium dodecylbenzene sulfonate (SDBS) was used in the experiment (Fangzheng Reagent Plant, Tianjin, China) to prepare the SAP-2 hydrogel. The mineral salts used to prepare aqueous solutions to determine the absorbability of the prepared hydrogels, such as cadmium acetate (II) dihydrate, copper (II) trihydrate, and nickel nitrate (II) hexahydrate, were supplied by (Merck). Hydrochloric acid, used to desorb the heavy metal ions loaded on hydrogels, was prepared using adsorption experiments developed by Sigma-Aldrich.

### 4.2. Preparation of the Hydrogels

The hydrogels used in the experiment were prepared as follows:

Preparing the gel SAP-1: The pullulan was dissolved with distilled water by placing it in a round-bottomed flask. The flask was placed in an oil bath at 60 °C. Potassium persulfate was added to the solution with continuous stirring for 10 min.

The crosslinker N,N-methylene bisacrylamide (MBA) was added to acrylic acid and acrylamide monomers to form (pullulan-g-poly (AA-co-AAm)) according to the proportions indicated in Table 4. The solution was left for 12 h in an oil bath; then, the hydrogel was placed in distilled water to remove the soluble parts for 24 h in a shaker at room temperature. The hydrogel was dried for 48 h under vacuum at 37 °C [43,46].

Preparation of SAP-2 gel: The high-content anionic surfactant with cleaning, wetting, foaming, emulsifying, and dispersing properties, such as (SDBS), according to the proportions indicated in Table 1, was added immediately after dissolving the pullulan with distilled water by placing it in a round-bottomed flask. Then, the same procedure was repeated for the preparation of SAP-1 [43,46].

### 4.3. Characterization Techniques

A Fourier transform infrared (FT-IR) test (Shimadzu, Kyoto, Japan) with a range of 400–4000 cm^−1^ was conducted using KBr pellets to determine the chemical composition of the prepared gels. The morphological properties of the samples’ surfaces were studied using the Field Emission Scanning Electron Microscopes (FE-SEM) technique (Zeiss Model ULTRA Plus, Jena, Germany). Hydrogen-1, nuclear magnetic resonance (^1^H NMR) (Bruker BioSpin GmbH, Rheinstetten, Germany) was used to identify the functional aggregates of hydrogels. An atomic absorption spectrometer (AAS-7000, Shimadzu, Kyoto, Japan) was used to estimate the heavy metal ions remaining in the aqueous solution after the prepared hydrogels adsorbed some of them.

### 4.4. Adsorption and Desorption of Metal Ions

#### 4.4.1. Adsorption Experiments

##### Adsorption Capacity and Removal Efficiency

A study of adsorption experiments was conducted to determine the ability of the prepared hydrogels to adsorb heavy metal ions such as cadmium acetate (II) dihydrate, copper (II) trihydrate, and nickel (II) hexahydrate from their aqueous solutions. A total of 0.02 g of dried hydrogels were dissolved in 2 mL of distilled water for 1 h at room temperature and a shaker was used to increase the absorption process. A total of 100 mL of the heavy metal ions presented above, with a concentration of 300 mg/L and a pH of 5.87, 5.76, and 5.34, respectively. A total of 10 mL of the above metal ion solutions was added to the swollen hydrogels for 24 h at room temperature and they were placed on a shaker to improve the absorption of the aqueous metal solutions. The experiments were repeated three times for each metal ion, and the adsorption capacity was calculated according to the following equation [65]:

(1) $$q_{t}=\frac{C_{0}-C_{t}}{m}\times V$$

where ($q_{t}$) is the adsorption capacity at time (*t*) (mg/g), *m* is the mass of hydrogel (g), *V* is the volume of metal ion solution in L, and $C_{0}$ and $C_{t}$ are the initial concentration and the concentration at time (*t*) in mg/L, respectively. The adsorption uptake at equilibrium ($q_{e}$, mg/g) and removal efficiency (%) were calculated using the following equation [66]:

(2) $$q_{e}=\frac{C_{0}-C_{t}}{m}\times V$$

(3) $$Removal(\%)=\frac{C_{0}-C_{e}}{C_{0}}\times 100$$

where $C_{e}$ is the equilibrium concentration (mg/L).

##### Kinetics Ions Adsorption

To determine the heavy metal ions’ adsorption efficiency by the hydrogels, the linear form of pseudo-first-order and pseudo-second-order, respectively, are presented in the following equations [34]:

(4) $$\log\left(q_{e}-q_{t}\right)=logq_{e}-\frac{K_{1}}{2.303}t$$

(5) $$\frac{t}{q_{t}}=\frac{1}{K_{2\;}q_{e}^{2}}-\frac{t}{q_{e}}$$

where *K*_1_ (min^−1^) and *K*_2_ (g.mg^−1^, min^−1^) are the adsorption rate constants of the pseudo-first- and second-order models, respectively. To investigate the adsorption mechanism more accurately, the intraparticle diffusion model can be used according to the Weber–Morris kinetic model presented in the following equation [66]:

(6) $$q_{t}=K_{id}t^{0.5}+C$$

where *C* is the intercept and *K_i_* (mg g^−1^ min^0.5^) is the intra-particle diffusion rate constant.

#### 4.4.2. Desorption Experiments

To detect the desorption of heavy metal ions loaded on hydrogels, experiments were conducted on the adsorption of ions from aqueous solutions by hydrogels. The hydrogels loaded with heavy metal ions were dried under vacuum for 24 h at 37 °C. Then, 10 mL of hydrochloric acid was added at a concentration of 0.1 M. The hydrogels were then placed in a shaker to increase the desorption of metal ions from the hydrogels for 24 h at room temperature. The desorption of metal ions from the hydrogel was calculated according to Equation (2). The desorption efficiency was also calculated according to the following equation [34]:

(7) $$Efficiency\left(\%\right)=\frac{C_{d}}{C_{a}}\times 100$$

where *C_d_* and *C_a_* are the desorbed and adsorbed metal amounts in mg/g, respectively.

#### 4.4.3. Reusability Experiments

Adsorption experiments with heavy metal ions loaded on hydrogels were conducted by repeating the adsorption–desorption cycles three times. The hydrogel loaded with ions was immersed in the desorption medium (hydrochloric acid) for 24 h. The hydrogel was filtered, washed with distilled water several times, dried in an oven, and reused for the next cycle. Hydrochloric acid solutions loaded with heavy metal ions were sent to an atomic absorption spectrometer (AAS-7000, Shimadzu, Kyoto, Japan) to determine the ion concentration.

## Figures and Tables

**Table 1:** Results of experimental and theoretical equilibrium capacity with kinetic rate constants of metal ions at different adsorption models.

Samples	Metal Ions	A1 (mg/g) ^a^	A			B			C		
			A2 (mg/g) ^b^	K1 (min^−1^)	R^2^	A3 (mg/g) ^c^	K2 (g. mg^−1^, min^−1^)	R^2^	Ki1 Mg. g^−1^ min^−0.5^	Ki2 Mg. g^−1^ min^−0.5^	Ki3 Mg. g^−1^ min^−0.5^
SAP-1	Cd (II)	146.9	156	0.0019	0.88	164.20	1.79 × 10^−5^	0.98	3.66	3.54	1.54
	Ni (II)	126.75	130.72	0.002	0.87	137.36	3.18 × 10^−5^	0.98	3.88	3.54	1.50
	Cu (II)	154	167.01	0.0023	0.86	168.63	2.50 × 10^−5^	0.96	3.30	3.28	0.51
SAP-2	Cd (II)	190.722	195.68	0.0027	0.95	206.61	2.74 × 10^−5^	0.99	6.60	4.29	1.14
	Ni (II)	179.94	189.41	0.0018	0.93	201.21	1.39 × 10^−5^	0.98	4.48	3.90	1.28
	Cu (II)	187.978	206.03	0.0024	0.92	214.59	1.47 × 10^−5^	0.98	5.0	4.88	1.22

A1: q e(experimental); A2: q e(calculated) for pseudo-first-order model; A3: q e(calculated) for pseudo-second order model; A: pseudo-first-order model; B: pseudo-second-order model; C: intra-particle diffusion model, R^2^: correction factor; K1, K2: pseudo-first- and second-order kinetic rate constants Ki1, Ki2, Ki3: intra-particle diffusion rate constants for first, second, and third cycles. ^a–c^ The standard deviation around the mean of A1, A2, and A3 for SAP-1 and SAP-2 was <10% and <5% respectively.

**Table 2:** Chemical elements remain in hydrogel (SAP-2) after the desorption of heavy metal ions for each cycle.

Elements	Desorption of Cd (II)			Desorption of Ni (II)			Desorption of Cu (II)		
	A	B	C	A	B	C	A	B	C
C	40.76	42.59	45.72	54.14	45.87	44.18	48.84	51.7	49
N	12.19	13.1	10.03	11.55	14.15	15.05	13	14.65	14.56
O	30.36	30.92	37.09	25.36	34.55	33.05	27.11	28.04	32.76
Si	2.1	1.97	1.92	0.62	0.76	0.8	1.69	0.95	1.05
Cl	1.84	1.3	2.13	2.11	0.98	4.06	1.74	1.97	0.57
Al	1.22	1.56	1.5	2.11	2.07	2	1.7	1.01	1.41
Cu	-	-	-	-	-	-	5.92	1.68	0.65
Ni	-	-	-	4.11	1.62	0.86	-	-	-
Cd	11.53	8.56	1.61	-	-	-	-	-	-

A: Wt.% of first cycle; B: Wt.% of second cycle; C: Wt.% of third cycle.

**Table 3:** Comparison of the maximum adsorption capacities of metal ions by various adsorbent hydrogels in the literature and this study.

Hydrogel	Metal Ions	Adsorption Capacity (mg/g)	Ref.
Hydrogel–biochar composite	Cd (II)	63.58	[62]
Cellulose hydrogel	Cu (II)	52.30	[63]
Chitosan–PVA composite hydrogel	Cu (II)	62.10	[64]
Pullulan-g-poly(AA-co-Aam) hydrogel	Cd (II)	169.79	[43]
NaAlg-g-P(AA-co-AM) hydrogel, obtained via SDBS micelle templating	Cd (II) Ni (II) Cu (II)	31.18 6.720 67.99	[46]
PUL-g-P(AA-co-AM) hydrogel	Cd (II) Ni (II) Cu (II)	146.90 126.75 154.00	This study
PUL-g-P(AA-co-AM) hydrogel, obtained via SDBS micelle templating	Cd (II) Ni (II) Cu (II)	190.72 179.94 187.98	This study

**Table 4:** The composition ratios of Pullulan-based hydrogels.

Samples	Pullulan	Monomers		MBA (Crosslinker)	K_2_S_2_O_8_ (Initiator)	Surfactant (SDBS)
		Acrylic Acid	Acrylamide			
SAP-1	17%	35%	35%	7%	6%	-
SAP-2	17%	34%	34%	7%	6%	2%

## Data Availability

The original contributions presented in the study are included in the article, further inquiries can be directed to the corresponding authors.

## References

[1] Sinha V., Chakma S. (2019). Advances in the preparation of hydrogel for wastewater treatment: A concise review. J. Environ. Chem. Eng..

[2] Sultan M., Abdelhakim A.A., Nassar M. (2021). Cu_2_O-mediated assembly of electrodeposition of Au nanoparticles onto 2D metal-organic framework nanosheets for real-time monitoring of hydrogen peroxide released from living cells. Anal. Bioanal. Chem..

[3] Korde J.M., Kandasubramanian B. (2020). Naturally biomimicked smart shape memory hydrogels for biomedical functions. Chem. Eng. J..

[4] Li Y., Zhang H., Ma C., Yin H., Gong L., Duh Y., Feng R. (2019). Durable, cost-effective and superhydrophilic chitosan-alginate hydrogel coated mesh for efficient oil/water separation. Carbohydr. Polym..

[5] Kandry G., Aboelmagd E.I., Ibrahim M.M. (2019). Cellulosic-based hydrogel from biomass material for removal of metals from waste water. J. Macromol. Sci. Part A Pure Appl. Chem..

[6] Sugumaran K.R., Ponnusami V. (2017). Review on production, downstream processing and characterization of microbial pullulan. Carbohydr. Polym..

[7] Singh R.S., Kaur N., Kennedy J.F. (2019). Pullulan production from agro-industrial waste and its applications in food industry: A review. Carbohydr. Polym..

[8] Abdulkadhim M.K., Habeeb S.A. (2022). The possibility of producing uniform nanofibers from blends of natural biopolymers. Mater. Perform. Charact..

[9] Wan G., Cheng Y., Song J., Chen Q., Chen B., Liu Y., Ji S., Chen H., Wang Y. (2020). Nucleus-targeting near-infrared nanoparticles based on TAT peptide-conjugated IR780 for photo-chemotherapy of breast cancer. Chem. Eng. J..

[10] Yenchit S., Tadokoro Y., Iwamori S. (2019). Measuring active oxygen species across a nonwoven fabric using a pullulan-mixed methylene blue thin film and electron spin resonance. IEEJ Trans. Sens. Micromach..

[11] Tabasum S., Noreen A., Maqsood M.F., Umar H., Akram N., Nazli Z.-I.-H., Chatha S.A.S. (2018). A review on versatile applications of blends and composites of pullulan with natural and synthetic polymers. Int. J. Biol. Macromol..

[12] Saber-Samandari S., Gulcan H.O., Saber-Samandari S., Gazi M. (2014). Efficient removal of anionic and cationic dyes from an aqueous solution using pullulan-graft-polyacrylamide porous hydrogel. Water Air Soil Pollut..

[13] Singh R., Pal D., Mathur A., Singh A., Krishnan M.A., Chattopadhyay S. (2019). An efficient pH sensitive hydrogel, with biocompatibility and high reusability for removal of methylene blue dye from aqueous solution. React. Funct. Polym..

[14] Bhadani B., Abe M. (2016). Structural diversity, physicochemical properties and application of imidazolium surfactants: Recent advances. Adv. Colloid Interface Sci..

[15] Siyal A.A., Shamsuddin M.R., Low A., Rabat N.E. (2020). A review on recent developments in the adsorption of surfactants from wastewater. J. Environ. Manag..

[16] Bakshi M.S., Kaur I. (2003). Aggregates of cationic surfactants and anionic polyelectrolytes influenced by bulky head group modifications. Colloids Surf. A Physicochem. Eng. Asp..

[17] Pal P., Pal A. (2017). Enhanced Pb^2+^ removal by anionic surfactant bilayer anchored on chitosan bead surface. J. Mol. Liq..

[18] Harutyunyan L.R., Harutyunyan R.S., Gabrielyan G.A., Lasareva E.V. (2019). Modification of chitosan and chitosan succinate by surfactants and investigation of their properties. Colloids Surf. A Physicochem. Eng. Asp..

[19] Pal P., Pal A. (2017). Surfactant-modified chitosan beads for cadmium ion adsorption. Int. J. Biol. Macromol..

[20] Chatterjee S., Lim S.R., Woo S.H. (2011). Adsorption of a cationic dye, methylene blue, onto chitosan hydrogel beads generated by anionic surfactant gelation. Environ. Technol..

[21] Kongarapu R.J., Nayak A.K., Khobragade M.U., Pala A. (2018). Surfactant bilayer on chitosan bead surface for enhanced Ni (II) adsorption. Sustain. Mater. Technol..

[22] Vakili M., Rafatullah M., Ibrahim M.H., Abdullah A.Z., Salamatinia B., Gholami Z. (2016). Chitosan hydrogel beads impregnated with hexadecylamine for improved reactive blue 4 adsorption. Carbohyd. Polym..

[23] Shaban S.M., Aiad I., Moustafa A.H., Aljoboury O.H. (2019). Some alginates polymeric cationic surfactants; surface study and their evaluation as biocide and corrosion inhibitors. J. Mol. Liq..

[24] Das D., Pal A. (2016). Adsolubilization phenomenon perceived in chitosan beads leading to a fast and enhanced malachite green removal. Chem. Eng. J..

[25] Kumar V., Parihar R.D., Sharma A., Bakshi P., Sidhu G.P.S., Bali A.S., Karaouzas I., Bhardwaj R., Thukral A.K., Gyasi-Agyei Y. (2019). Global evaluation of heavy metal content in surface water bodies: A meta-analysis using heavy metal pollution indices and multivariate statistical analyses. Chemosphere.

[26] Olapido A.A., Gazi M. (2015). Nickel removal from aqueous solutions by alginate-based composite beads: Central composite design and artificial neural network modelling. J. Water Process Eng..

[27] Zimta A.-A., Schitcu V., Gurzau E., Stavaru C., Manda G., Szedlacsek S., Berindan-Neagoe I. (2019). Biological and molecular modifications induced by cadmium and arsenic during breast and prostate cancer development. Environ. Res..

[28] Li Y., Shen R., Wu H., Yu L., Wang Z., Wang D. (2020). Liver changes induced by cadmium poisoning distinguished by confocal Raman imaging. Spectrochim. Acta Part A Mol. Biomol. Spectrosc..

[29] Reyes-Hinojosa D., Lozada-Perez C.A., Cuevas Y.Z., Lopez-Reyes A., Martinez-Nava G., Fernandez-Torres J., Olivos-Meza A., Landa-Solis C., Guiterrez-Ruiz M.C., del Castillo E.R. (2019). Toxicity of cadmium in musculoskeletal diseases. Environ. Toxicol. Pharmacol..

[30] Araujo C.V.M., Pontes J.R.S., Blasco J. (2019). Does the previous exposure to copper alter the pattern of avoidance by zebrafish in a copper gradient scenario? Hypothesis of time-delayed avoidance due to pre-acclimation. Sci. Total Environ..

[31] da Costa J.S., Bertizzolo E.G., Bianchini D., Fajardo A.R. (2021). Adsorption of benzene and toluene from aqueous solution using a composite hydrogel of alginate-grafted with mesoporous silica. J. Hazard. Mater..

[32] Ji Z., Zhang H., Zhang Y., Chen T., Long Z., Li M., Pei Y. (2019). Distribution, ecological risk and source identification of heavy metals in sediments from the Baiyangdian Lake, Northern China. Chemosphere.

[33] Salimi A., Rahimi H.-R., Foroontanfar H., Jafari E., Ameri A., Shakibaie M. (2019). Toxicity of microwave-assisted biosynthesized zinc nanoparticles in mice: A preliminary study. Artif. Cells Nanomed. Biotechnol..

[34] Saber-Samandari S., Gazi M. (2015). Pullulan based porous semi-IPN hydrogel: Synthesis characterization and its application in the removal of mercury from aqueous solution. J. Taiwan Inst. Chem. Eng..

[35] Barakat M.A. (2011). New trends in removing heavy metals from industrial wastewater. Arab. J. Chem..

[36] Efome J.E., Rana D., Matsuura T., Lan C.Q. (2019). Effects of operating parameters and coexisting ions on the efficiency of heavy metal ions removal by nano-fibrous metal-organic framework membrane filtration process. Sci. Total Environ..

[37] Nemati M., Hosseini S.M., Shabanian M. (2017). Novel electrodialysis cation exchange membrane prepared y 2-acrylamido-2-methylpropane sulfonic acid; heavy metal ions removal. J. Hazard. Mater..

[38] Areco M.M., Saleh-Medina L., Trinello M.A., Marco-Brown J.L., Alfonso M.D.S. (2013). Adsorption of Cu(II), Zn(II), Cd(II) and Pb(II) by dead *Avena fatua* biomass and the effect of these metals on their growth. Colloids Surf. B Biointerfaces.

[39] Radi S., El Abiad C., Moura N.M.M., Faustino M.A.F., Neves M.G.P.M.S. (2019). New hybrid adsorbent based on porphyrin functionalized silica for heavy metals removal: Synthesis, characterization, isotherms, kinetics and thermodynamics studies. J. Hazard. Mater..

[40] Ramos-Lara F., Ramírez M.Q., Flores M., Arroyo R., Caldiño U. (2006). Optical spectroscopy of Nd^3+^ ions in poly (acrylic acid). J. Phys. Condens. Matter.

[41] Tao X., Xie Y., Zhang Q., Qiu X., Yuan L., Wen Y., Li M., Yang X., Tao T., Xie M. (2016). Cholesterol-modified amino-pullulan nanoparticles as a drug carrier: Comparative study of cholesterol-modified carboxyethyl pullulan and pullulan nanoparticles. Nanomaterials.

[42] Chen L., Wang X., Ji F., Wang Y.B.J., Wang X., Guo L., Li Y. (2015). New bifunctional-pullulan-based micelles with good biocompatibility for efficient co-delivery of cancer-suppressing p53 gene and doxorubicin to cancer cells. RSC Adv..

[43] Sonmez B., Celikkol A.N. (2021). Pullulan based hydrogels for the removal of various metal ions from aqueous solutions. J. Environ. Chem. Eng..

[44] Chang H., Wang G., Yang A., Tao X., Liu X., Shen Y., Zheng Z. (2010). A transparent, flexible, low-temperature, and solution-processible graphene composite electrode. Adv. Funct. Mater..

[45] Mukwada L.T., Mochane M.J., Motaung T.E., Motloung S.V., Koao L.F. (2017). Effect of sodium dodecylbenzene sulphonate modifier and PP-g-MA on the morphology and thermal conductivity of PP/EG composites. Plast. Rubber Compos..

[46] Tally M., Atassi Y. (2016). Synthesis and characterization of pH-sensitive superabsorbent hydrogels based on sodium alginate-g-poly (acrylic acid-co-acrylamide) obtained via an anionic surfactant micelle templating under microwave irradiation. Polym. Bull..

[47] Repo E., Warchol J.K., Kurniawan T.A., Sillanpaa M.E.T. (2010). Adsorption of Co(II) and Ni(II) by EDTA- and/or DTPA-modified chitosan: Kinetic and equilibrium modeling. Chem. Eng. J..

[48] Kutsevol N.V., Zheltonozhskaya T.B., Demchenko O.V., Kunitskaya L.R., Syromyatnikov V.G. (2004). Effect of the structure of poly(vinyl alcohol)-graft-polyacrylamide copolymers on their thermooxidative stability. Polym. Sci. Ser. A.

[49] Luo M.-T., Li H.-L., Huang C., Zhang H.-R., Xiong L., Chen X.-F., Chen X.-D. (2018). Cellulose-based absorbent production from bacterial cellulose and acrylic acid: Synthesis and performance. Polymers.

[50] Bian Y., Bian Z., Zhang J., Ding A., Liu S., Zheng L., Wang H. (2015). Adsorption of cadmium ions from aqueous solutions by activated carbon with oxygen-containing functional groups. Chin. J. Chem. Eng..

[51] Ratnamala G.H., Shetty K.V., Srinikethan G. (2012). Removal of remazol brilliant blue dye from dyecontaminated water by adsorption using red mud: Equilibrium, kinetic, and thermodynamic studies. Water Air Soil Pollut..

[52] Doshi B., Ayati A., Tanhaei B., Repo E., Sillanpää M. (2018). Partially carboxymethylated and partially cross-linked surface of chitosan versus the adsorptive removal of dyes and divalent metal ions. Carbohyd. Polym..

[53] Ni X., Li Z., Wang Y. (2018). Adsorption Characteristics of Anionic Surfactant Sodium Dodecylbenzene Sulfonate on the Surface of Montmorillonite Minerals. Front. Chem..

[54] Zeng L., Zhou F., Hu R., Xie W., Wang G., Yang C., Liang E., Xu W., Van der Bruggen B. (2020). Synthesis of cross-linked carboxyl modified polyvinyl alcohol and its application in selective adsorption separation of Cu(II) from Cd(II) and Ni(II). J. Polym. Environ..

[55] Lucaci A.R., Bulgariu D., Popescu M.-C., Bulgariu L. (2020). Adsorption of Cu(II) ions on adsorbent materials obtained from marine red algae *Callithamnion corymbosum* sp.. Water.

[56] Xie R., Jin Y., Chen Y., Jiang W. (2017). The importance of surface functional groups in the adsorption of copper onto walnut shell derived activated carbon. Water Sci. Technol..

[57] Bafrooee A.A.T., Panahi H.A., Hasani A.H., Moniri E., Miralinaghi M. (2020). Removal of Hg^2+^ by carboxyl-terminated hyperbranched poly(amidoamine) dendrimers grafted superparamagnetic nanoparticles as an efficient adsorbent. Environ. Sci. Pollut. Res..

[58] Guo X., Li M., Liu A., Jiang M., Niu X., Liu X. (2020). Interfacial adhesion of polylactic acid on cellulose surface: A molecular dynamics study. Water.

[59] Zhang L., Song F., Wu Y., Cheng L., Qian J., Wang S., Chen Q., Li Y. (2018). A novel amino and carboxyl functionalized mesoporous silica as an efficient adsorbent for nickel (II). J. Chem. Eng. Data.

[60] Serra R.S., Molina-Mateo J., Torregrosa-Cabanilles C., Andrio-Balado A., Duen J.M.M., Serrano-Aroca Á. (2020). Bio-nanocomposite hydrogel based on zinc alginate/graphene oxide: Morphology, structural conformation. Polymers.

[61] Chen X., Zhou S., Zhang L., You T., Xu F. (2016). Adsorption of heavy metals by graphene oxide/cellulose hydrogel prepared from NaOH/urea aqueous solution. Materials.

[62] Li S., Chen G. (2018). Using hydrogel-biochar composites for enhanced cadmium removal from aqueous media. Mater. Sci. Eng. Int. J..

[63] Teow Y.H., Kam L.M., Mohammad A.W. (2018). Synthesis of cellulose hydrogel for copper (II) ions adsorption. J. Environ. Chem. Eng..

[64] Song Q., Gao J., Lin Y., Zhang Z., Xiang Y. (2020). Synthesis of cross-linking chitosan-PVA composite hydrogel and adsorption of Cu(II) ions. Water Sci. Technol..

[65] Habeeb S.A., Nadhim B.A. (2023). Removal of nickel (II) ions, low-level pollutants, and total bacterial colony count from wastewater by composite nanofiber film. Sci. Iran..

[66] Khoshkho S.M., Tanhaei B., Kazemi A.A.M. (2021). Preparation and characterization of ionic and non-ionic surfactants impregnated κ-carrageenan hydrogel beads for investigation of the adsorptive mechanism of cationic dye to develop for biomedical applications. J. Mol. Liq..

